# Data Analytics of Electronic Health Records to Enhance Care of Coronary Artery Disease in Younger Women with Avoiding Possible Delay in Treatment

**DOI:** 10.3233/SHTI220277

**Published:** 2022-06-06

**Authors:** Maryam Panahiazar, Andrew M. Bishara, Yorick Chern, Roohallah Alizadehsani, Omar S. Latif, Dexter Hadley, Ramin E. Beygui

**Affiliations:** a Department of Surgery, Division of Cardiothoracic Surgery, School of Medicine, University of California San Francisco, San Francisco, CA, USA; b Department of Anesthesia and Perioperative Care, University of California San Francisco, San Francisco, CA, USA; c Department of Bioengineering, University of California Berkeley, Berkeley, CA, USA; d Institute for Intelligent Systems Research and Innovation, Deakin University, Australia; e Department of Clinical Sciences, College of Medicine, University of Central Florida, Orlando, FL, USA; f Department of Internal Medicine, Mayo Clinic, Rochester, MN, USA; g Bakar Computational Health Sciences Institute, School of Medicine, University of California San Francisco, San Francisco, CA, USA

**Keywords:** Electronic Health Records, Coronary Artery Disease, Data Science

## Abstract

Early detection plays a key role to enhance the outcome for Coronary Artery Disease. We utilized a big data analytics platform on ~32,000 patients to trace patients from the first encounter to CAD treatment. There are significant gender-based differences in patients younger than 60 from the time of the first encounter to Coronary Artery Bypass Grafting with a p-value=0.03. This recognition makes significant changes in outcome by avoiding delay in treatment.

## Introduction

Coronary Artery Disease (CAD), is the leading cause of death, morbidity, and mortality in the United States and globally. Women are reported to have a poorer outcome than men. However, in both genders, discrepancies in risk factors and outcomes within CAD diagnosis and treatment have been well reported [1, 2, 4]. The reasons for these discrepancies have not been fully addressed. Underlying risk factors for cardiovascular disease (CVD) in general have different physiological effects on the two genders. Our systematic review of gender-based studies of diagnosis and treatments of CAD over the last 20 years [3] demonstrated multiple significant studies exist in targeting gender-based discrepancies. The complete causes of these discrepancies and the effect age hold treatment delay in age groups have yet to be fully elucidated and require further analysis to design interventions and structures to combat bias. Identification of women in an earlier stage of CAD could have a significant impact on patients' health and outcome in later stages. This may significantly reduce unnecessary treatments, and cost, and potentially mitigate the morbidity and mortality of downstream procedures associated with incorrect or late diagnosis of CAD.

## Methods

The data were selected from the de-identified Electronic Health Record (EHR) from a total of ~1m patients admitted to UCSF between 2011 and 2018. The following cohort search criteria were developed to select the study cohort for this research: Coronary Artery Disease (CAD), based on ICD10 code (120-125). Patients with missing values specifically for the ICD10 code were excluded. Patients defined as unknown, unspecified, and nonbinary gender definitions were excluded. Those included were patients who met the above criteria, leading to a cohort size of 32,000 patients with CAD. This dataset consisted of de-identified patient ID and diagnosis based on the ICD10 code. Patient characteristics include age, gender, BMI, blood pressure, cholesterol, ethnicity, outcomes such as stroke, and status, smoking are selected and defined with the average and/or percentage as shown in Table 1.

There were no significant differences in the comorbidities of the selected cohort except COPD that was ~3% more in women. For procedure codes, we used Current Procedural Terminology (CPT) and date of procedure services for both invasive and noninvasive procedures including CABG, EKG, stress test, echocardiogram, Diagnostic Cardiac Catheterization (D_Cath), and Therapeutic Cardiac Catheterization (T_Cath/PCI). We included the medication code, medication name, and date of the medication orders. The patients whose medical history does not include at least one element from the set of CPT codes were eliminated from the initial cohort of patients. We designed and implemented a time-series data analytics platform in our previous research and specified the platform for this study. We traced patients from the initial encounter at UCSF medical center who have then been prescribed a new medication or subsequently underwent an invasive or noninvasive cardiac related procedure. Next, we calculated the age of patients at time points they received relevant medication or procedure. The time interval between events has been calculated. In the next step, the time between the sequence of events for each patient and a group of similar patients in different age groups was calculated. Event time was defined as the date of the first event until the date of the next event. We explored all possible existing sequences of event paths (e.g. D_Cath => CABG) for individual patients in each age group.

Then we searched for evidence that there are gender-based differences in different age groups. The first step to validate this hypothesis is to determine the first point of an encounter with a physician when a patient was suspected of having cardiovascular disease. We included the treatment path with suspicion of potential cardiovascular disease that combined both procedures and medications. Any type of medication that belongs to the classes of cardiovascular and cardiac drugs, anticoagulants, antiplatelets, aspirin, beta-blockers, statins are included as the starting point for medication so called “first event” for patients suspected to be at risk for cardiovascular disease. Then, both the men and women datasets are merged, on the same paths of the event for each age group (<60, 60-80, >80) defined by expert and ready for validation of hypothesis.

All data are extracted with MySQL queries from original datasets at UCSF medical center. For feature selection (e.g. choose specific data points and features such as including smoking condition and dropping marriage status for this research, and common medication selection for creating the dictionary of medication, we used deep learning with the help of TensorFlow and libraries such as Kibana. We made a specific class of Artificial neural network (ANN) called a Multi-Layer Perceptron (MLP) [6]. We made 3 layer MLP with Keras. Input layers for the data, hidden layers to control the ANN inner workings like parameters, the column in the training set, dropout Layers to control overfitting. As a result, we choose the characteristics which are shown in table one. Some features (e.g. marriage status, height) had been removed because of less priority in the model to predict the patient with CAD disease as a target.

## Results and Validation

**Figure 1.**
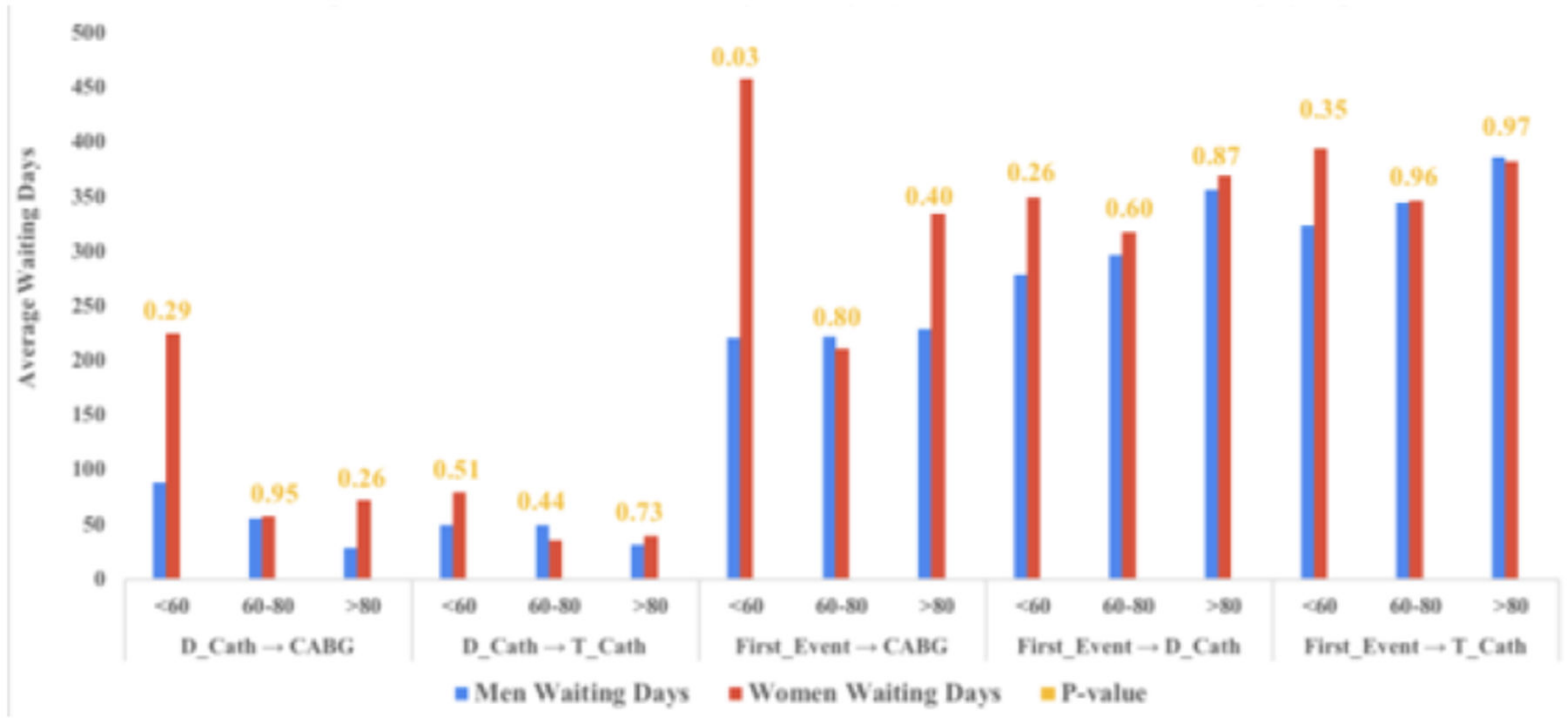
Statistical analysis results. It illustrates gender comparison for average waiting days between events in different age

We validated our data in Python to find possible differences in each age group in different steps of the diagnosis and treatment. 2 sample t-Tests are performed for each of the three age groups (<60, 60-80, >80) to test whether the population means of each two groups are equal or not. P-value calculated (considering 95% Confidence Interval). ANOVA test was performed for the comparison of age groups within the 3 women and men patient groups. As shown in Figure 1, there are statistically significant gender-based differences for waiting days between events in patients younger than 60 from the time of the first encounter to eventual CABG with a p-value=0.03 which is less than 0.05 as we defined (95%CI). There is no significant difference for patients between 60 and 80 (p-value=0.8) and older than 80 (p-value=0.4). In summary for women younger than 60 from the time of very first encounter (e.g. prescribing Aspirin, B-blocker) takes an average of max of 460 days to have a CABG procedure versus for men in the same age group is 220 days (240 days longer).

## Conclusions

In this study, we explored the Electronic Health Record at UCSF to prove the hypothesis of the existence of different waiting times (delay) between different steps of diagnosis and treatment of CAD in women versus men in different age groups. We believe early detection at a younger age could prevent unexpected events, better outcome and avoid extra costs.

## Figures and Tables

**Table 1 –:** Characteristics. It shows Characteristics (#12134 women and #20761 men) with CAD diseases

Characteristics (%)	Men	Women
Never Smoked	63.24%	54.19%
Never Smoker but Passive Smoke Exposure	0.43%	0.73%
Former Smoker	45.46%	30.14%
Current Every Day Smoker	5.91%	4.58%
Ethnicity (Hispanic or Latino)	8.36%	10.63%
Ethnicity (Not Hispanic or Latino )	82.97%	82.46%
Stroke	14.25%	35.15%
Status (alive)	88.70%	88.88%
Status (deceased)	10.10%	10.17%
Characteristics (Avg.)	Men	Women
Age	68.87	68.12
BMI	29.38	34.10
Blood Pressure (Diastolic Blood Pressure)	70.94	68.97
Blood Pressure (Systolic Blood Pressure)	129.8	131
Cholesterol (Total)	156.9	180.4
Cholesterol (LDL)	85.46	98.35
Cholesterol (HDL)	47.15	57
